# Characterization of the human Activin-A receptor type II-like kinase 1 (*ACVRL1*) promoter and its regulation by Sp1

**DOI:** 10.1186/1471-2199-11-51

**Published:** 2010-06-29

**Authors:** Eva M Garrido-Martin, Francisco J Blanco, Africa Fernandez-L, Carmen Langa, Calvin P Vary, Ursula E Lee, Scott L Friedman, Luisa M Botella, Carmelo Bernabeu

**Affiliations:** 1 Centro de Investigaciones Biológicas (CIB), Consejo Superior de Investigaciones Cientificas (CSIC) and Centro de Investigación Biomédica en Red de Enfermedades Raras (CIBERER), Ramiro de Maeztu 9, 28040 Madrid, Spain; 2 Department of Cancer Biology and Genetics, Memorial Sloan-Kettering Cancer Center, New York, NY, USA; 3 Center for Molecular Medicine, Maine Medical Center Research Institute, Scarborough, ME, USA; 4 Division of Liver Diseases. Mount Sinai School of Medicine, New York, NY, USA

## Abstract

**Background:**

Activin receptor-like kinase 1 (ALK1) is a Transforming Growth Factor-β (TGF-β) receptor type I, mainly expressed in endothelial cells that plays a pivotal role in vascular remodelling and angiogenesis. Mutations in the ALK1 gene (*ACVRL1*) give rise to Hereditary Haemorrhagic Telangiectasia, a dominant autosomal vascular dysplasia caused by a haploinsufficiency mechanism. In spite of its patho-physiological relevance, little is known about the transcriptional regulation of *ACVRL1*. Here, we have studied the different origins of *ACVRL1 *transcription, we have analyzed *in silico *its 5'-proximal promoter sequence and we have characterized the role of Sp1 in the transcriptional regulation of *ACVRL1*.

**Results:**

We have performed a 5'Rapid Amplification of cDNA Ends (5'RACE) of *ACVRL1 *transcripts, finding two new transcriptional origins, upstream of the one previously described, that give rise to a new exon undiscovered to date. The 5'-proximal promoter region of *ACVRL1 *(-1,035/+210) was analyzed *in silico*, finding that it lacks TATA/CAAT boxes, but contains a remarkably high number of GC-rich Sp1 consensus sites. In cells lacking Sp1, *ACVRL1 *promoter reporters did not present any significant transcriptional activity, whereas increasing concentrations of Sp1 triggered a dose-dependent stimulation of its transcription. Moreover, silencing Sp1 in HEK293T cells resulted in a marked decrease of *ACVRL1 *transcriptional activity. Chromatin immunoprecipitation assays demonstrated multiple Sp1 binding sites along the proximal promoter region of *ACVRL1 *in endothelial cells. Furthermore, demethylation of CpG islands, led to an increase in *ACVRL1 *transcription, whereas *in vitro *hypermethylation resulted in the abolishment of Sp1-dependent transcriptional activation of *ACVRL1*.

**Conclusions:**

Our results describe two new transcriptional start sites in *ACVRL1 *gene, and indicate that Sp1 is a key regulator of *ACVRL1 *transcription, providing new insights into the molecular mechanisms that contribute to the expression of *ACVRL1 *gene. Moreover, our data show that the methylation status of CpG islands markedly modulates the Sp1 regulation of *ACVRL1 *gene transcriptional activity.

## Background

ALK1 (Activin receptor-Like Kinase 1) is a transmembrane type I receptor of the Transforming Growth Factor-β (TGF-β) superfamily of ligands, mainly found in endothelial cells. Its expression has been reported not only in highly vascularized tissues including lung, placenta, and heart [1,2], but also at specific sites of epithelial-mesenchymal interactions [3], and in other cell types such as monocytes [4], microglia [5], skin fibroblasts [6], stellate hepatic cells [7], chondrocytes [8], neural crest stem cells [9] and more recently myoblasts [10]. Nonetheless, most studies to date suggest that its major roles are related to the endothelial specific expression pattern. ALK1 is involved in angiogenesis [11,12], and there is growing evidence indicating that it plays a key function in the arterial/venous differentiation during embryonic vascular development [13,14]. It has been reported that ALK1 interacts with three ligands: with TGF-β1 and TGF-β3, in complex with the receptor type II (TβR-II) [15]; and with Bone Morphogenetic Protein 9 (BMP9), in complex with the Activin Receptor type IIA (ActRIIA) or the BMP receptor type II (BMPRII) [16]. In the endothelium, circulating TGF-β signals from the lumen of the vascular vessel to the cytoplasm of the endothelial cell by interacting with its specific receptor complex. This complex consists of three different dimeric proteins: receptor type II (TβR-II), receptor type I (TβR-I) and an ancillary co-receptor (TβR-III: Betaglycan or Endoglin) [17]. ALK5 is the predominant TβR-I in the majority of the cell types, but in endothelial cells ALK1 shares the TβR-I function with ALK5 *in vitro*. The significance of this apparent redundancy is explained because ALK1 and ALK5 signal in opposite directions, balancing the TGF-β signalling pathway in this cell type [11]. ALK5 is able to arrest the cell growth, leading to a differentiated state in the maturation phase of angiogenesis, with formation of new extracellular matrix around the new vessel formed. ALK1 appears to play opposite physiological functions, since it is responsible for the events occurring during the activation phase of angiogenesis, including metalloprotease activation, proliferation of endothelial cells, and inhibition of differentiation [18]. Thus, these complementary effects are mediated through different target genes of the two signalling pathways. ALK5 signals through Smad2/3 to regulate *PAI-1 *(Plasminogen Activator Inhibitor-1), *Collagen I*, or *NOS-3/eNOS *(Endothelial Nitric Oxide Synthase), whereas ALK1 signals through Smad1/5/8 to induce genes involved in proliferation such as *Id1 *(Inhibitor of differentiation 1), *Id2 *(Inhibitor of differentiation 2), *Smad6*, *Smad7*, *ENG *(Endoglin) or *BMPRII *[19]. In addition, it has been shown that BMP9 is a quiescence factor for the microvasculature [20].

The gene encoding ALK1 (*ACVRL1*, Activin-A receptor type II-like kinase 1) spans 15,943 bp within the large arm of chromosome 12 (12q11-q14), coded on the positive strand (GeneID: 94). *ACVRL1 *cDNA was described first in 1993 simultaneously by two different groups showing a characteristic transcriptional start site (TSS), where the first exon is transcribed but not translated. To date, two different variants of mRNA transcripts have been described: variant 1 [GenBank:NM_000020.2] [21] and variant 2 [GenBank:NM_001077401.1] [22], both encoding the same ALK1 protein of 503 amino acids.

Mutations in *ACVRL1 *give rise to an autosomal vascular dysplasia called Hereditary Haemorrhagic Telangiectasia type 2 (HHT2) [1], while HHT type 1 is caused by mutations in *ENG*, the gene coding for the TGF-β co-receptor Endoglin [23]. HHT1 and HHT2 are genetically dominant and mutant homozygosis is lethal, as confirmed in knock out mice for *ENG *and *ACVRL1 *[24-26]. Current estimates suggest that one in 5,000-8,000 people are affected by HHT [27]. Patients are clinically diagnosed according to the Curaçao criteria [28], including nose bleeds, mucocutaneous telangiectases, internal arteriovenous malformations and familial inheritance. Around 90% of HHT patients have been genetically diagnosed as HHT1 or HHT2, and multiple mutations along exonic regions of *ACVRL1 *and *ENG *have been described [29]. Because approximately 10% of HHT patients clinically diagnosed have unidentified mutations, the study of intronic sequences, splice sites and promoter regions of both genes is of critical importance. Moreover, haploinsufficiency is currently accepted as the basis for the pathogenicity of HHT [29] and therefore the understanding of the transcriptional mechanisms and the assessment of ways to increase the transcription rate may be crucial to identify strategies to counteract haploinsufficiency. While several reports have already described the organization and control of *ENG *promoter [30-33], very little has been done in relation to the *ACVRL1 *promoter. In this work, we have analyzed the 5'-proximal *ACVRL1 *promoter region, characterized new transcriptional initiation points, and analyzed the transcriptional mechanisms that regulate its expression.

## Results

### Identification of novel TSSs for human *ACVRL1 *gene revealed by 5'RACE

**Figure 1:**
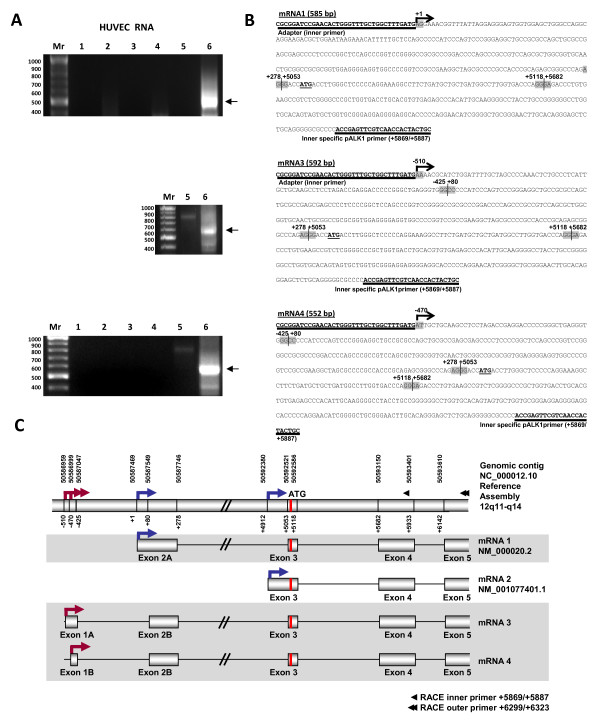
**5' Rapid Amplification of cDNA Ends (5'RACE) of *ACVRL1 *transcripts from HUVECs**. Electrophoretic analysis of nested PCR amplification products. Lanes: 1 and 2 (without template; outer and inner nested PCRs), 3 and 4 (RNA without CIP/TAP treatment; outer and inner), 5 and 6 (CIP/TAP treated RNA; outer and inner). More than 10 different experiments were performed and three representative gels are shown.** (B) **Nucleotide sequence of the products. Primers used are underlined. Grey boxes indicate the junctions between different exons. The predominant transcript found in HUVECs corresponds to the mRNA1 previously described in placenta. Two novel variants described in this work have been named mRNA3 [GenBank:HM161905] and mRNA4 [GenBank:HM161906]. Numbers are given according to the genomic sequence from + 1 TSS. Fragments starting from -510 of mRNA3 and from -470 of mRNA4 are the newly observed transcribed regions. The sequences of these three isoforms are identical downstream from + 79. **(C) **Schematic representation of the 5' flanking region of *ACVRL1 *transcripts. The previously described mRNA1 [GenBank:NM 000020.2] and mRNA2 [GenBank:NM 001077401.1], and mRNA3 and mRNA4 found in this work are shown. All *ACVRL1 *encoded proteins found to date have the same 503 amino acid sequence. mRNA1 has been found with the same characteristics as previously described. Taking into account the new sequence, *ACVRL1 *rearranges from 10 to 11 exons; starting the new exon 1 at -510 (variant 1A) or -470 (variant 1B). Also, previously described exon 1 is renamed as exon 2 (variants 2A and 2B). Red arrows represent the two newly described TSSs. Blue arrows represent the two TSSs previously known.

In order to characterize the origins of *ACVRL1 *transcription in endothelial cells, a 5'RACE of total RNA obtained from HUVECs was performed. After nested PCR, the amplified bands were cloned and sequenced (Figure 1A and 1B). The three major products amplified correspond to three different 5'flanking regions. More than 50% of the clones corresponded to the mRNA1 of *ACVRL1 *previously described [21], whereas two new TSSs (mRNA3 and mRNA4) were also identified in several clones from independent experiments (Figure 1B). Interestingly, both alternative mRNAs begin the transcription upstream the TSS (+ 1), in the positions -510 and -470, respectively. Accordingly, the exons have been renumbered in this work in such a way that the new exon 1 is upstream of the previously described TSS (+ 1). The numbering of the TSS (+ 1) has been maintained because it appears to be the predominant TSS. Thus, the previously considered exon 1 turns into exon 2, being named 2A, in the case of NM_000020.2 and 2B in the case of NM_001077401. Likewise, the newly discovered upstream exon now becomes exon 1, named 1A in the mRNA3, or 1B, in the variant mRNA4.

Figure 1C shows the scheme of all the *ACVRL1 *transcript variants found in this work (mRNA1, 3 and 4) and in previous reports (mRNA1 and 2). The variant mRNA1 begins in the position + 1, followed by an exon of 278 bp (exon 2A) and then upon splicing of the longest intron (~ 5 kb) of *ACVRL1*, it continues with an exonic sequence containing the ATG start codon (exon 3). The mRNA2 has no exon 2A, and its exon 3 is longer in its 5'region, containing an additional 141 bp fragment. The starting ATG is located at the beginning of exon 3 (+ 5,058/+5,060), coinciding with mRNA1, therefore, both transcripts give rise to the same 503 amino acid protein.

The fragments obtained in the present work correspond to isoforms mRNA3 and mRNA4. The corresponding cDNA sequences have been deposited in GenBank database with accession numbers HM161905 and HM161906, respectively. Both of them reveal a cryptic exon placed upstream the + 1, resulting in two, instead of one, transcribed but not translated exons. This event gives rise to a rearrangement from 10 to 11 exons in the composition of the coding region of *ACVRL1*.

### The human *ACVRL1 *promoter lacks TATA/CAAT boxes and has multiple Sp1 motifs

**Figure 2:**
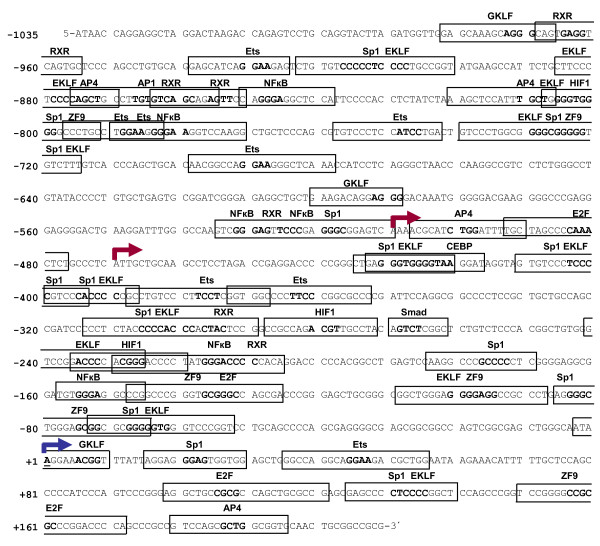
**Nucleotide sequence of the 5'-flanking region of the *ACVRL1 *gene and *in silico *analysis of the putative transcription factor binding sites**. Red arrows show the newly described TSSs (-510 and -470). The blue arrow indicates the already described TSS (+ 1). The sequence lacks TATA and CAAT boxes. Putative transcription factor binding motifs for AP1 (Activator protein 1), AP4 (Activator protein 4), CEBP (CCAAT/Enhancer Binding protein), E2F (Elongation Factor 2), EKLF/KLF1 (Krüppel-Like Factor 1), ETS (E26-Transformation Specific transcription factor), GKLF/KLF4 (Krüppel-Like Factor 4), HIF (Hypoxia Inducible Factor), NFκB (Nuclear Factor of kappa light polypeptide gene enhancer in B-cells), RXR (Retinoid X Receptor), Smad (Sma and Mad-related factor), Sp1 and ZF9/KLF6 (Krüppel-Like Factor 6) are indicated by boxes. The core consensus sequences for each factor are in bold. A large number of Sp1 consensus binding sites are present in the sequence. Data were generated using the Genomatix MatInspector software tool.

Human *ACVRL1 *gene resides on chromosome 12q11-q14 [34]. Thus, a 1,244 bp fragment comprising from position -50,586,434 to position -50,587,679 of the contig [GenBank:NC_000012.10, Reference assembly] was amplified by PCR from human genomic DNA (Figure 2). This fragment corresponds to positions from -1,035 bp to + 210 bp relative to the (+ 1) TSS. Interestingly, this 5'-proximal regulatory region of *ACVRL1 *lacks TATA and CAAT boxes. A predictive *in silico *analysis of *ACVRL1 *promoter using the Genomatix MatInspector software tool revealed a very high number of GC-rich regions and multiple putative Sp1 binding consensus sites (Table 1). Fourteen different possible sites for Sp1 interaction were found, located in both strands: Forward strand (Sites -810/-796, -734/-720, -728/-714, -524/-510, -435/-421, -88/-74, -70/-56 and + 15/+30); and reverse strand (Sites -919/-905, -411/-397, -401/-387, -309/-295, -184/-170 and + 124/+139). All of these sites have a very high similarity with the theoretical matrix (score > 0.85). Three AP1 and four AP4 sites were found dispersed along the sequence. In addition, the 5'-flanking sequence of *ACVRL1 *gene contains several putative regulatory elements also present in other endothelial genes (see Figure 2 and Table 1). These consensus elements include eight motifs for the Ets (E26-Transformation-Specific) family of transcription factors, 23 sequences sensitive for KLF (Krüppel-Like Factor) recognition, seven NFκB (Nuclear Factor kappa-light-chain-enhancer of activated B cells) motifs, five E2F (Elongation Factor 2) consensus sequences, one Smad binding element (SBE) and seven sites for RXR (Retinoid X Receptor) dimers. Moreover, three HIF (Hypoxia Inducible Factor) consensus sequences were found distributed along the *ACVRL1 *promoter sequence.

**Table 1:** Main putative transcription factor binding sites found in the ACVRL1 promoter region

Family	Start pos.	End pos.	Str.	Matrix sim.	Core sim.	Family	Start pos.	End pos.	Str.	Matrix sim.	Core sim.
**AP1**	-875	-855	-	0.823	1.0	**HIF**	-808	-792	+	0.85	0.9
	-873	-853	+	0.899	0.813		-286	-274	+	0.885	1.0
	-871	-851	-	0.994	1.0		-236	-219	-	0.843	0.75

**AP4**	-881	-865	-	0.923	1.0	**NFKB**	-851	-839	+	0.901	1.0
	-820	-804	-	0.87	0.882		-786	-774	+	0.852	1.0
	-506	-490	-	0.961	1.0		-784	-772	-	0.919	1.0
	+179	+195	-	0.855	1.0		-534	-522	+	0.985	1.0
						
**CEBP**	-432	-418	+	0.945	0.971		-533	-521	-	0.947	1.0
						
**EKLF/KLF1**	-918	-902	-	0.924	1.0		-220	-208	+	0.84	1.0
	-886	-870	-	0.911	1.0		-158	-146	+	0.838	1.0
						
	-808	-792	+	0.975	1.0	**RXR**	-979	-955	+	0.954	1.0
	-737	-721	+	0.932	1.0		-871	-847	+	0.758	0.771
	-731	-715	+	0.917	1.0		-864	-840	+	0.818	0.824
	-433	-417	+	0.977	1.0		-535	-511	+	0.847	0.952
	-410	-394	-	0.933	1.0		-441	-417	+	0.763	0.812
	-405	-389	-	0.95	1.0		-314	-290	-	0.819	0.81
	-313	-297	-	0.974	1.0		-230	-206	-	0.791	0.905
						
	-241	-225	-	0.916	1.0	**SMAD**	-270	-262	+	0.969	1.0
						
	-107	-91	+	0.923	1.0	**SP1**	-919	-905	-	0.901	0.807
	-73	-57	+	0.916	1.0		-810	-796	+	0.885	0.872
	-68	-52	+	0.971	1.0		-734	-720	+	0.997	1.0
	+125	+141	-	0.947	1.0		-728	-714	+	0.879	1.0
						
**ETS**	-939	-919	+	0.961	1.0		-524	-510	+	0.913	1.0
	-797	-777	+	0.922	1.0		-435	-421	+	0.881	0.872
	-791	-771	+	0.965	1.0		-411	-397	-	0.89	0.807
	-758	-738	-	0.943	1.0		-401	-387	-	0.895	0.872
	-699	-679	+	0.972	1.0		-309	-295	-	0.891	0.872
	-388	-368	-	0.945	1.0		-184	-170	-	0.98	1.0
	-373	-353	-	0.914	1.0		-88	-74	+	0.885	1.0
	+36	+56	+	0.927	1.0		-70	-56	+	0.892	0.872
						
**E2F**	-493	-477	+	0.849	1.0		+15	+30	+	0.987	1.0
	-143	-127	-	0.861	0.81		+124	+139	-	0.93	0.877
						
	+98	+114	-	0.971	1.0	**ZF9/KLF6**	-815	-793	-	0.891	0.923
	+99	+115	+	0.964	1.0		-739	-717	-	0.899	1.0
	+150	+166	-	0.875	1.0		-148	-126	-	0.881	1.0
						
**GKLF/KLF4**	-981	-969	+	1.000	0.862		-109	-87	-	0.897	0.821
	-600	-588	+	1.000	0.883		-86	-64	-	0.879	1.0
	-3	+9	+	0.770	0.868		+150	+172	+	0.936	1.0

Note: Start/End pos, starting/ending position of the consensus site in the sequence; Str, Strand-sense; Matrix sim, Matrix (groups of functionally similar transcription factors) similarity factor (0-1); Core sim, Core consensus sequence (4 highest conserved positions) similarity factor (0-1); AP1, Activator Protein 1; AP4, Activator Protein 4; CEBP, CCAAT/Enhancer Binding Protein; EKLF, Erythroid Krüppel-Like Factor; ETS, E26-Transformation-Specific transcription factor; E2F, Elongation Factor 2; GKLF, Gut-related Krüppel-Like Factor; HIF, Hypoxia Inducible Factor; NF?B, Nuclear Factor of kappa light polypeptide gene enhancer in B-cells; RXR, Retinoid X Receptor; SMAD, Sma and Mad-related; SP1, Specificity Protein 1; ZF9, Krüppel-like Zinc Finger protein 9.

### The *ACVRL1 *promoter region is highly conserved among different species

**Figure 3:**
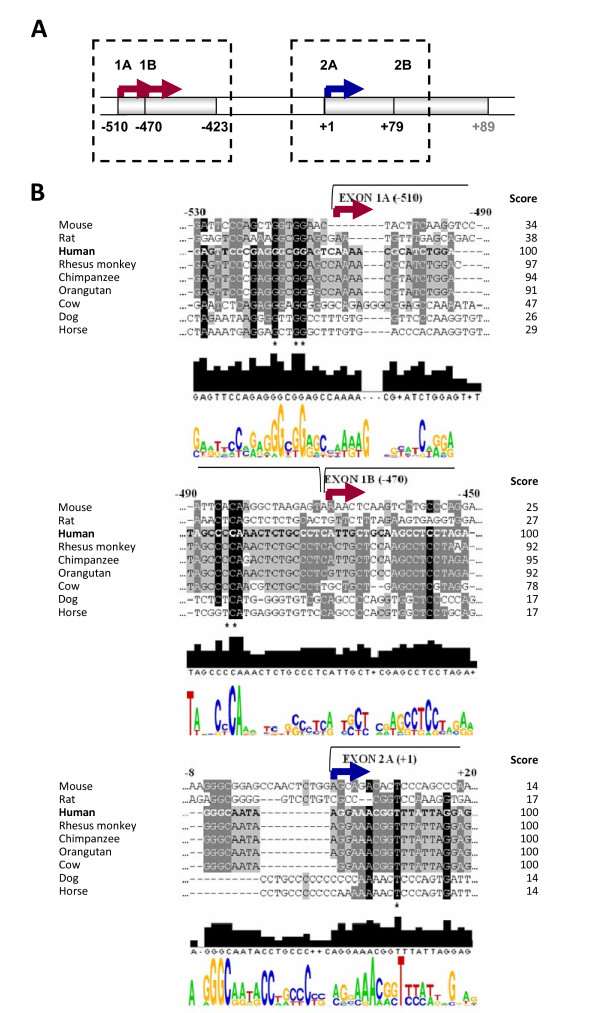
**Sequence alignment of the human *ACVRL1 *TSSs across different mammalian species**. **(A) **Scheme of the human sequences used for the alignment. The blue arrow indicates the previously described TSS (+ 1). The red arrows indicate the new TSSs identified in Figure 1. **(B) **Sequences from chimpanzee, orangutan, rhesus monkey, cow, dog, horse, mouse and rat were compared with the human -1,035/+210 sequence. Sequences were obtained from NCBI-GenBank and EMBL databases (see Methods). Asterisks indicate the totally conserved residues across species. Alignments of the putative regulatory sites are shown with a colored background based on the identity between species. The degree of homology is: black > dark grey > light grey. Score means percentage of identity with the human sequence. Brackets indicate exonic sequences.

The *ACVRL1 *proximal promoter sequence has several features that suggest a high level of regulation. One of these features is the possibility to begin the transcription in different points. To evaluate the importance of each putative regulatory site, the *ACVRL1 *promoter of several mammalian species were compared using the ClustalW2 algorithm. The species analyzed included mouse, rat, cow, orangutan, rhesus monkey, horse, chimpanzee and dog. For the alignments, we selected the same region than the one we were studying in humans (from -1,035 bp to + 210 bp of the TSS). Figure 3 shows the comparative alignment of the *ACVRL1 *human sequence surrounding the different TSSs with the same regions in other animal species. Panel A shows a scheme of the regions aligned in panel B. The previously described TSS (identified by the + 1) has a highly conserved sequence among primates (AGGAAACGG), suggesting an important functional role. Moreover, the two newly described TSSs are conserved only in this group of species (-510 bp AAAACGC in exon 1A and -470 bp ATTGCT in exon 1B). Of note, both sequences at the beginning of exons 1A and 1B are less conserved than the region surrounding TSS (+ 1).

**Figure 4:**
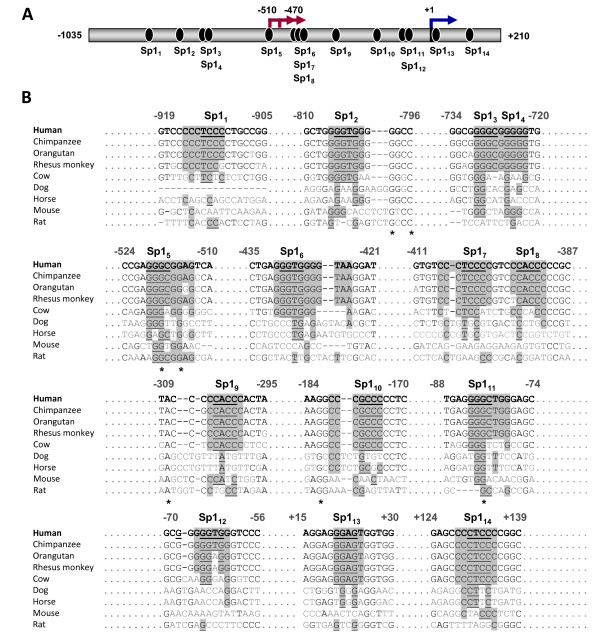
**Sp1 consensus binding sites located along the human *ACVRL1 *promoter and alignment with other mammalian species**. **(A) **Schematic representation of the 14 Sp1 consensus sites found *in silico *along the -1,035/+210 fragment of *ACVRL1 *promoter sequence. Red arrows show the newly described TSSs. The blue arrow indicates the already described TSS (+ 1). **(B) **Alignment with other mammalian species (source of sequences as in Figure 3). Numbers are referred to the human sequence and range from -1,035 to + 210 bp. Asterisks indicate the totally conserved residues across species. Alignments of the putative regulatory sites are shown in grey background. All the human nucleotides selected for the alignment (grey background) have a Ci-value > 60. Ci = Consensus index value. It represents the degree of conservation of each position within the matrix. Putative core sequences for the binding of Sp1 are underlined. The core sequence (usually 4 residues) of a matrix is defined as the most conserved with the theoretical consensus. Additional information about each site can be found in Table 1.

Another interesting feature about the putative *ACVRL1 *promoter is the high number of Sp1 consensus sites along the sequence, and near to the TSSs. These sites are important as possible key regulators for *ACVRL1 *transcription, so we compared these motifs among different species. The scheme of Sp1 sites present in the sequence is shown in Figure 4A. The alignments of these fragments and the same regions from other animal species are included in Figure 4B. Of note, these sites appear highly conserved, especially between humans and other primates, which is a characteristic of promoters under Sp1 regulation and lacking TATA/CAAT boxes.

### Basal activity of *ACVRL1 *promoter suggests the presence of possible positive and negative regulatory regions

**Figure 5:**
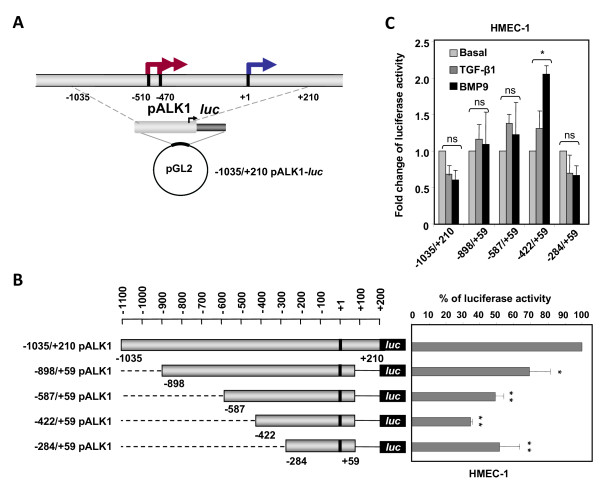
**Transcriptional activity of the human *ACVRL1 *promoter**. **(A) **Schematic representation of the *ACVRL1 *promoter fragment cloned into the promoterless pGL2-*luc *reporter vector with the three TSS. **(B) **Left, 5'-deleted construct series of the whole sequence obtained by PCR and cloned into pGL2-*luc*. The size of each construct compared with the size of the whole promoter construct is shown in the scheme. Right, transient transfection of *ACVRL1 *promoter 5'-deleted constructs in the human endothelial cell line HMEC-1. Transfection efficiency was corrected by relating luciferase activity to β-galactosidase activity. Results are expressed as a percentage of activity respect to the activity of the full length construct (-1,035/+210; 100%) (*p < 0.05, **p < 0.01 versus -1,035/+210 pALK1 construct). **(C) **Effect of ALK1 ligands TGF-β1 and BMP9 on *ACVRL1 *promoter activity. HMEC-1 cells were transiently transfected with *ACVRL1 *5'-deleted mutants, and pretreated with 1 ng/ml TGF-β1 for 3 hours or 0.5 ng/ml BMP9 for 15 hours. Results are shown in fold induction values respect to basal activity. No significant effect on *ACVRL1 *promoter activity was observed, except a little increase with BMP9 on the -422/+59 construct (*p < 0.05; **p < 0.01; ns = not significant).

To assess the transcriptional activity of the *ACVRL1 *promoter fragment, it was cloned into a luciferase reporter vector. A schematic representation of the *ACVRL1 *promoter fragment cloned into pGL2-*luc*, with the locations of the different TSS and the ATG translation start codon, is shown in Figure 5A. Serial 5'-deleted constructs from -1,035 bp to -284 bp of the *ACVRL1 *proximal promoter were generated (Figure 5B, left) and their basal transcription activity assessed in HMEC-1. In this endothelial cell line, the shortest construct maintains the majority of the transcriptional activity, whereas the -422/+59 construct displays one third of the activity compared with the whole sequence (Figure 5B, right). In order to unravel if *ACVRL1 *could be transcriptionally regulated by its own ligands, the effect of TGF-β1 or BMP9 was assessed. HMEC-1 cells were transfected with different pALK1 reporter constructs and treated with either TGF-β1 or BMP9 using the time and dose previously established in endothelial cells [35]. None of these treatments had a significant effect on the transcriptional activity of the reporter constructs, except for the construct -422/+59, whose basal activity seems to be slightly upregulated in the presence of BMP9 (Figure 5C).

### Sp1 is critical for the *ACVRL1 *basal transcription

**Figure 6:**
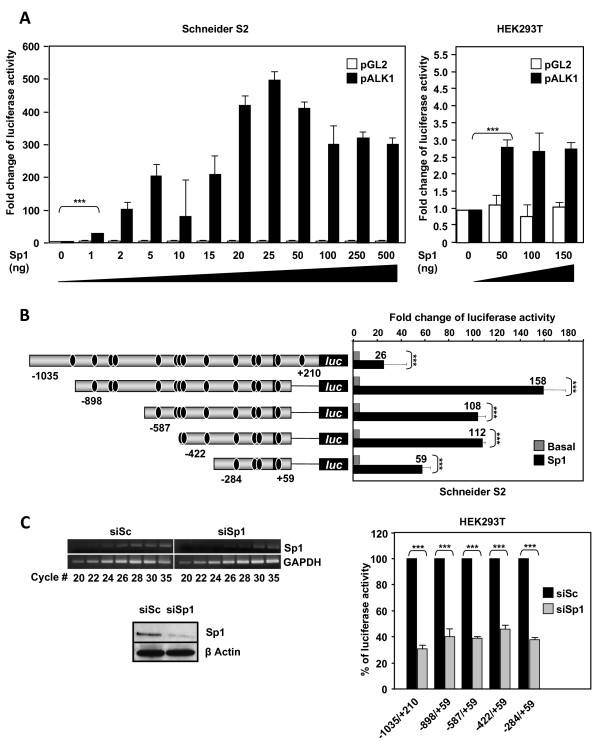
**Effect of Sp1 expression on *ACVRL1 *promoter activity**. **(A) **Dose-response effect of Sp1 on the transcriptional activity of *ACVRL1 *promoter in Schneider S2 and HEK293T cells. S2 (Sp1-less) and HEK293T cells were cotransfected with the pGL2 empty vector or the *ACVRL1 *promoter construct -1,035/+210 and with increasing amounts of the Sp1 expression vector (pPac-Sp1 and pCIneo-Sp1, respectively). Luciferase activity was corrected with β-galactosidase activity and expressed as fold induction of the transcriptional activity of pALK1 in the absence of exogenous Sp1. **(B) **Left, scheme showing the distribution of the different Sp1 consensus binding sites along the *ACVRL1 *promoter (black ovals) in the different constructs. Right, transient transfection of Schneider S2 cells with 25 ng of pPac-Sp1 and the indicated *ACVRL1 *promoter constructs. Fold-induction values respect to basal activity are indicated on top of each bar. **(C) **Effect of Sp1-knock down on *ACVRL1 *transcriptional activity. HEK293T cells were transfected with Sp1 siRNA. Left, Sp1 mRNA and protein levels were measured by semiquantitative RT-PCR and western blot after 48 hr. Right, 24 hr after the siRNA Sp1 transfection, the different *ACVRL1 *promoter constructs were transfected. The transcriptional activity of all the fragments was measured and normalized by the β-galactosidase activity. Basal pALK1 activity (100%) and the reduction after Sp1 silencing (grey bars) are shown. In every case, Sp1 suppression resulted at least in a decrease of 50% in *ACVRL1 *transcriptional activity (***p < 0.005).

Since 14 putative target sequences for Sp1 were predicted in the *ACVRL1 *promoter, their contribution to the basal activity was assessed in Schneider *Drosophila *embryonic S2 cells. These cells are undifferentiated and do not express endogenous Sp1 or any other related member of its family. As shown in Figure 6A, left, the proximal promoter of *ACVRL1 *shows background levels of basal activity in the absence of Sp1. By contrast, ectopic expression of Sp1 shows a dramatic effect on pALK1 activity. Even with very low amounts of the transfected Sp1 expression vector, the basal transcription of pALK1 is increased ~ 100 fold following a dose-response curve. There is a saturating effect around 25 ng Sp1 where the promoter is stimulated ~500 fold. The same experiment performed in HEK293T cells resulted in the stimulation of the basal activity up to 3-fold (Figure 6A, right). This moderate increase, as compared to S2 cells (~500 fold), is likely due to the endogenous expression of Sp1 in HEK293T cells. Thus, small amounts of Sp1 expression are sufficient to saturate all the Sp1 sites in the *ACVRL1 *promoter, leading to the maximum transcriptional activity that the promoter can achieve.

The next step was to dissect the Sp1 transcription induction in the different promoter constructs. S2 cells were co-transfected with Sp1 and with the different 5'-deleted constructs of the reporter pALK1 (Figure 6B). All constructs showed low basal transcriptional activities, which have been referred to value 1 and the stimulation is expressed as fold induction. When as little as 25 ng of the Sp1 expression vector was transfected, the activity levels of each construct were remarkably stimulated (between 26- to 158-fold induction values). It is worth mentioning that the maximum activity reached by the -422/+59 construct did not parallel its relatively low activity in endothelial cells, which have basal Sp1 levels (Figure 5B). This discrepancy is probably explained by the different background of transcription factors present in *Drosophila *embryonic versus human HMEC-1 cells.

To further assess the relevance of Sp1 in *ACVRL1 *transcription, Sp1 expression was abolished in mammalian Sp1-expressing cells. Thus, HEK293T cells transfected with siRNA targeted to Sp1 led to a marked decrease (more than 50%) of the *ACVRL1 *transcriptional activity in all the constructs (Figure 6C). These data confirm that Sp1 is essential for the transcriptional activity of the *ACVRL1 *promoter.

### Chromatin immunoprecipitation of endogenous Sp1 bound to *ACVRL1 *promoter in HUVECs

**Figure 7:**
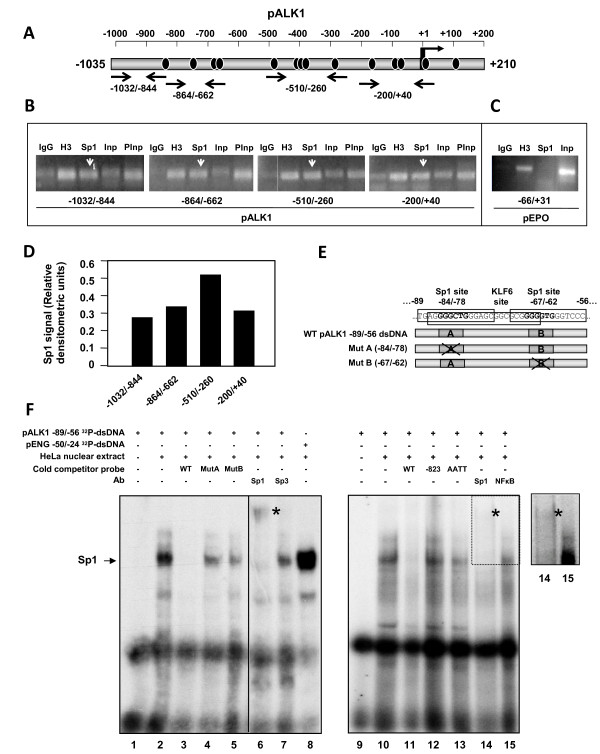
**Sp1 interacts with *ACVRL1 *promoter in HUVECs**. **(A) **Scheme showing the primers used for Chromatin immunoprecipitation (ChIP). The whole sequence of *ACVRL1 *promoter is mapped along four regions of approximately 200-250 bp. **(B) **Sp1 ChIP on *ACVRL1 *promoter in HUVECs. The chromatin was digested obtaining a 150-300 bp fragments-enrichment. Anti-Histone 3 and a pool of rabbit-IgGs were used as positive and negative controls. Input DNA was loaded before (Inp) and after (PInp) a preclearing process. **(C) **As a negative control of a gene promoter that does not ChIP with Sp1, a fragment of erythropoietin (*EPO*) promoter was used [36]. **(D) **Sp1 binding to the different pALK1 regions from the ChIP experiment in **B **was measured by densitometry of the individual bands and values of the (Sp1-IgG)/PInput ratios were represented. **(E) **Scheme of the *ACVRL1 *promoter fragment used as probe for EMSA assays and competitor mutant probes generated. **(F) **Electrophoretic Mobility Shift Assays (EMSA) shows the binding of Sp1 to the -89/-56 bp region of *ACVRL1 *promoter. Two Sp1 sites and one KLF6 site are framed in this region. EMSAs were performed with ^32^P-labelled WT probe. Cold probes were: WT; Mut A, mutated at the -84/-78 site; Mut B, mutated at the -67/-62 site; and two irrelevant sequences, the -823/-795 region of *ACVRL1 *promoter (-823) and AATT. A positive control of Sp1 was included in lane 8 using a probe from *ENG *promoter as described in Methods. The retarded Sp1 band is indicated by the arrow. The asterisks indicate the supershifted band obtained by addition of the anti-Sp1 antibody. The insert on the right, includes an over-exposition of the supershift corresponding to lanes 14 and 15. As negative controls, anti-Sp3 and anti-NFκB antibodies were included.

Because Sp1 is clearly able to stimulate the basal transcription of *ACVRL1 in vitro*, it was of interest to assess whether the physical binding of Sp1 to the *ACVRL1 *promoter occurs within the endothelial cells. For this purpose, a chromatin immunoprecipitation (ChIP) of endogenous Sp1 was performed in HUVECs. Four different regions were screened, spanning the whole -1,035/+210 bp sequence of *ACVRL1 *promoter (Figure 7A). PCR amplification yielded positive bands along the four fragments, suggesting that Sp1 is normally bound to all these fragments of the *ACVRL1 *promoter and, consequently, most of the putative theoretical binding sites are functionally active (Figure 7B). Densitometric analysis of the PCR enriched fragments revealed that all of them showed a clear Sp1 binding above background, yielding the strongest signal the fragment -510/-260 (Figure 7D). As a negative control, the erythropoietin (*EPO*) promoter, containing a Sp1 motif that recruits Sp1 only under hypoxic conditions [36], was used. In fact, the ChIP experiment demonstrated that Sp1 did not bind to this Sp1 site in the *EPO *promoter, confirming the specificity of the assay (Figure 7C).

Considering that the smallest fragment (-284/+59) maintains the majority of the promoter activity respect to the whole construct (Figure 5B), the nearest Sp1 sites upstream of the TSS (+ 1), appear to be critical for the *ACVRL1 *basal transcription. Interestingly, the -89/-56 fragment encompasses two adjacent Sp1 consensus elements, flanking a putative KLF6 binding site (Figure 7E). This fragment was selected as a probe for EMSA studies. A mobility shift appeared when nuclear extracts from HeLa cells (Sp1 rich) were incubated with the labelled probe and this binding was effectively competed with 100-fold excess of cold probe (Figure 7F). A supershift was observed in the presence of an antibody specific for Sp1 whereas anti-Sp3 or anti-NFκB antibodies did not affect the mobility shift, demonstrating the specificity for Sp1. The slight decrease of the retarded band in the presence of anti-Sp3 may be explained by the fact that Sp1 and Sp3 often bind to the same sites. Furthermore, single mutations of either Sp1 sites yielded retarded bands of a weaker intensity, suggesting that both sites are binding Sp1 independently. As negative controls, two Sp1 unrelated sequences, -823/-795 (containing consensus binding sites for other transcription factors, none of them homologous to Sp1 motifs) and AATT (double stranded poly dA-poly dT) were unable to compete with the probe, although with the AATT probe a slight non-specific reduction of the signal was observed.

### The methylation status modulates ALK1 expression in endothelial cells

**Figure 8:**
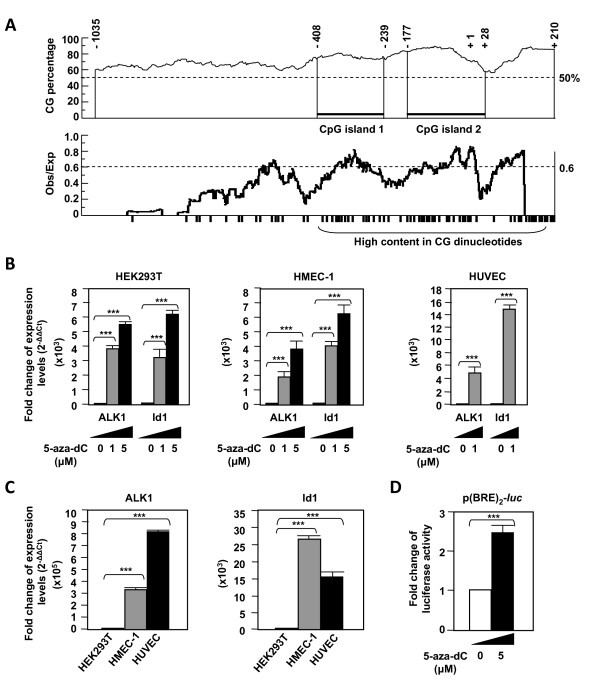
**Two CpG islands are present in the *ACVRL1 *promoter and treatment with the demethylating agent 5'-aza-2'-deoxycytidine increases ALK1 expression in endothelial cells**. **(A) **Schematic representation of the *ACVRL1 *promoter comprising the region -1,035 to + 210 bp. CG sites are depicted by black bars. Two CpG islands near the transcriptional start site were detected using CpGplot software tool. CG content is shown as percentage the total number of G+C (top), and by the methylation-susceptible CG pairs, represented by the observed-versus-expected index (bottom). **(B, C) **ALK1 and Id1 transcript levels from endothelial (HUVEC, HMEC-1) versus non endothelial (HEK293T) cells prior and after treatment with the demethylating agent 5-aza-dC. Id1 mRNA levels were measured as a target gene of ALK1 signalling. **(B) **Cells were treated with 1 μM or 5 μM 5-aza-dC for one week. Treatment with 5 μM was cytotoxic in HUVEC. RNA was extracted and mRNA levels were measured by real time RT-PCR. Results are shown as the fold change respect to basal expression (2 ^-ΔΔCt^).** (C) **Basal ALK1 and Id1 levels show the differences between ALK1 expression in endothelial cells HMEC-1 and HUVEC versus the HEK293T cells. **(D) **Effect of the demethylating agent 5-aza-dC on the ALK1 pathway specific reporter, p(BRE) _2_-luc, in HMEC-1 cells. Results are shown as fold change of expression levels or luciferase activity (***p < 0.005).

**Figure 9:**
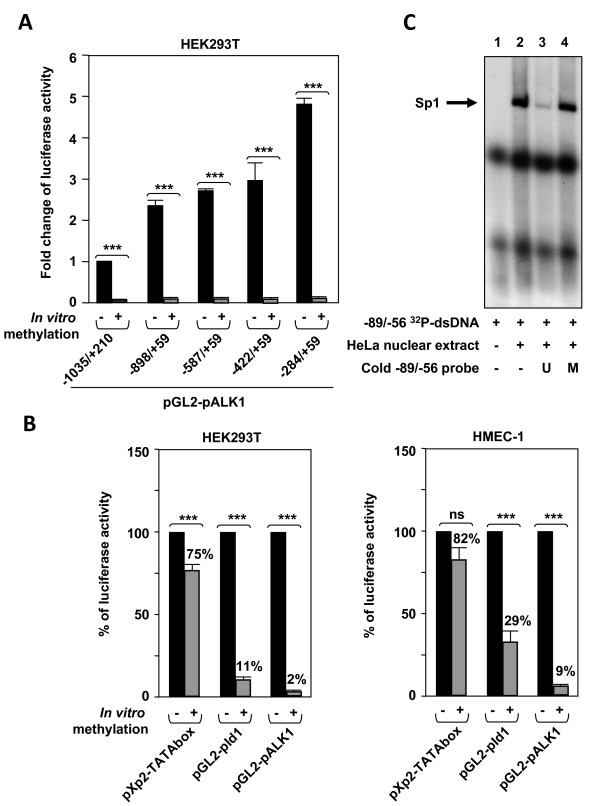
**Effect of *in vitro *methylation on the *ACVRL1 *promoter activity**. **(A, B) **Different reporter constructs were subjected to treatment with M.*Sss*I in the presence or absence of the substrate S-adenosyl-methyonine. Both mock-methylated and methylated constructs were transfected in HEK293T and HMEC-1 cells and the luciferase activity was measured. (***p < 0.005; ns = not significant). **(A) **Analysis of the different pALK1 reporter constructs. Results are shown as fold changes of luciferase activity **(B) **Analysis of a TATAbox minimal promoter (see Methods), the *Id1 *promoter construct and the -1035/+210 pALK1 reporter construct. Activities of untreated samples were given the arbitrary value of 100% and the activity of treated samples is indicated as percentage. **(C) **Electrophoretic mobility shift assay (EMSA) of the radiolabelled -89/-56 probe containing two Sp1 functional sites. Competition was carried out using the unmethylated (U) or the *in vitro *methylated (M) probe, as indicated.

*ACVRL1 *promoter showed several GC-rich regions in the *in silico *analysis of the -1,035/+210 bp fragment. Using CpGPlot software, two CpG islands were identified, based on their parameters: 1) CG percentage > 50%; and 2) a ratio of observed-to-expected > 0.6. These two islands comprise the regions between -408/-239 and -177/+28 (Figure 8A). Because these GC-rich regions are potential targets for Sp1 binding and methylation of cytosines in CpG islands is one of the main epigenetic modifications that regulate gene expression, we analyzed whether ALK1 expression could be influenced by promoter methylation. Indeed, as observed by real time PCR, when endothelial HMEC-1 and HUVEC cells were subjected to a treatment with the demethylating agent 5'-aza-2'-deoxycytidine (5-aza-dC), their ALK1 levels were upregulated up to ~4,000-fold (Figure 8B). Interestingly, HEK293T, a cell line that does not express ALK1 under normal conditions (Figure 8C), underwent ~6,000-fold activation of ALK1 expression after treatment with 5-aza-dC (Figure 8B). Likewise, Id1, which is a specific target gene of the ALK1/TGF-β1 signaling cascade activation, became upregulated in parallel to the increased levels of ALK1 in the three cell types (Figure 8B, C). Moreover, the activity of the reporter p(BRE)_2_-*luc*, specific of the ALK1 signalling pathway, was strongly enhanced upon treatment with 5-aza-dC. Conversely, the effect of *in vitro *hypermethylation was assessed. *ACVRL1 *promoter constructs were methylated *in vitro *with the CpG methyltransferase M.*Sss*I and the methylation status was checked by comparative digestion of both mock and methylated *ACVRL1 *promoter construct with *Hpa*II (data not shown). Then, HEK293T cells were transfected with mock-methylated or hypermethylated promoter constructs. As shown in Figure 9A the transcriptional activity of all the *ACVRL1 *constructs was completely abolished upon methylation. By contrast, the activity of the prolactin promoter region driven by a TATA box was almost unaffected (~80% versus untreated), whereas the activity of Id1 promoter construct was reduced at a much lower degree than the *ACVRL1 *construct (11% versus 2% of pALK1 in HEK293T; 29% versus 9% of pALK1 in HMEC-1) (Figure 9B). To assess whether the methylation status of the Sp1 motifs affected the Sp1 binding, the -89/-56 radiolabelled probe was competed with cold unmethylated and methylated probes. As shown in Figure 9C, the unmethylated probe clearly inhibited the Sp1 band, whereas the methylated probe was unable to compete. These results suggest that the methylation status of the promoter strongly modulates ALK1 expression.

## Discussion

The characterization of the 5'-proximal *ACVRL1 *promoter fragment has allowed us to identify two new transcripts, upstream of the previously published TSS. Those transcripts include an upstream elusive exon, meaning that *ACVRL1 *may have several TSSs. Thus, *ACVRL1*, rather than showing a unique transcription start site, contains different regions with a variable probability of transcription initiation, a characteristic of TATA-less genes. Interestingly, this is a common feature shared between *ACVRL1 *and *ENG*. It is tempting to speculate that the existence of different points of transcription initiation may allow a more flexible regulation depending on the tissue, or the developmental stage of the cell. In certain tissues like placenta, two main types of transcripts have been reported [21,22]. In HUVECs we have found two new transcripts that do not affect the open reading frame of the encoded protein. In the new transcripts, the first transcribed exon is not translated, and therefore no changes in the predicted protein sequence are observed. This observation emphasizes the importance of postranscriptional as well as transcriptional regulation mechanisms in the case of *ACVRL1*. The existence of different transcribed untranslated regions suggests regulation of ALK1 expression that involves the transcriptional rate, mRNA stability or interaction with other RNAs or RNA binding proteins. This feature is compatible with the high degree of conservation found among primates. Also, all the *ACVRL1 *transcripts described in human, orangutan, rhesus monkey and chimpanzee, seem to begin with an untranslated first exon. Thus, *ACVRL1 *may have important motifs for transcriptional regulation in the first exon. These motifs are included in the promoter fragment that has been subjected to study in this work.

Analysis of the basal promoter activity using luciferase-reporter experiments in a series of 5'-deleted mutant constructs revealed the existence of positive and negative regulatory elements. Interestingly, the shortest construct (-284/+59) displays a similar activity to that of the whole fragment (-1,035/+210). This finding suggests the existence of critical transcription factors binding to this area in order to enhance the transcriptional activity of the TATA-less *ACVRL1 *promoter. In agreement with this view, a high degree of conservation around the + 1 TSS among different species was found. The analysis also suggests the presence of a putative "repressor" segment between -422 and -284 positions, which inhibits *ACVRL1 *transcription. Interestingly, a similar region is found in the *ENG *promoter further upstream [30], something that deserves further investigation.

The *in silico *study of the *ACVRL1 *promoter sequence showed several important putative consensus elements, also found in *ENG *promoter [30,32] that could be critical for the coordinated transcriptional regulation of these genes. This finding suggests that genes involved in the same functional pathway are controlled in a similar manner. Thus, *ACVRL1 *may be regulated by some of the transcription factors also controlling *ENG *expression. In this sense, because *ENG *is regulated by HIF-1, Smad, KLF6, Ets, or Sp1 [31-33,37], it will be of interest to assess their functional implications in *ACVRL1*. Of note, three sequences matching HIF-1α motifs were found at positions -808/-792, -286/-274 and -236/-219 bp. The possibility that *ACVRL1 *could respond to HIF-1α is supported because hypoxia promotes angiogenesis, a process that is regulated by ALK1. In addition, multiple KLF consensus elements are dispersed along the *ACVRL1 *promoter region. Interestingly, this is another feature owing to the TGF-β receptor complex genes, in which the transcription is stimulated by KLF6 as an early injury response [18,32]. On the other hand, the presence of multiple Ets sites, suggests regulation by MAPKs (Mitogen Activated Protein Kinases) [38]. In this sense, in the functionally related gene, *ENG*, an Ets functional site was described in the proximal promoter [30]. We also note that the presence of AP1 and NFκB sites suggests that *ACVRL1 *could be a target for the inflammatory response. In contrast to the *ENG *proximal promoter that contains abundant SBE motifs, only a single putative SBE was found at -270 bp position of the *ACVRL1 *promoter. Also, at variance with *ENG*, the *ACVRL1 *promoter does not contain any TGF-β-responsive element (SBE) in the neighbourhood of Sp1 sites. Moreover, the TGF-β receptor type I (ALK5) gene (*TGFBRI)*, contains a SBE within the proximal promoter and both *ENG *and *TGFBRI *mRNA expression is upregulated by TGF-β [30,39]. Thus, the proximal region of *ENG *is regulated by TGF-β through Smads, and a direct cooperation Smad/Sp1 giving rise to an important increase in transcription [31]. Consistent with the lack of proximal responsive TGF-β elements within the *ACVRL1 *promoter, we found that its promoter activity was not regulated by TGF-β1 or BMP9. This does not exclude the possibility of a transcriptional regulation by TGF-β from SBEs placed further upstream, or by an indirect postranscriptional control through modifications of the receptor, which allow or prevent ALK1 to activate signalling through Smads. Addressing the functionality of all these transcription factors in the *ACVRL1 *promoter deserves an independent investigation. The present work has focused on the functional role of Sp1 on *ACVRL1 *basal transcription.

The most striking feature revealed by the *in silico *analysis of *ACVRL1 *is the presence of multiple Sp1 sites next to the different TSSs. Sp1 has been widely described as a general transcription factor involved in transcriptional basal mechanisms of gene promoters that lack TATA and CAAT core boxes. This is a shared characteristic among different housekeeping genes [40]. Interestingly, this is also the case of several TGF-β receptors such as *ENG *and *TGFBR1*, whose transcription is driven by Sp1 [30,31]. Further support for the involvement of Sp1 was obtained from the highly conserved alignment of the Sp1 consensus sites along the *ACVRL1 *promoter among several species that suggests a conserved biological relevance of Sp1 in the transcription of *ACVRL1*.

We have demonstrated here that Sp1 is a key factor necessary for the basal activation of *ACVRL1 *transcription. This effect has been observed both by Sp1 overexpression in cells lacking endogenous Sp1, and by interfering endogenous Sp1 with siRNA in mammalian cells. The large number of Sp1 consensus sites found in the *ACVRL1 *promoter, and the finding that small amounts of Sp1 can saturate its transcription, points out to a critical dependence on Sp1, and a fine tuning of *ACVRL1 *transcription by Sp1. Furthermore, a ChIP analysis in HUVECs after chromatin crosslinking revealed that Sp1 binds in vivo to the different consensus motifs along the whole proximal *ACVRL1 *promoter sequence studied (-1,032/+40). Thus, in endothelial primary cells growing in a rich medium, Sp1 may be binding to most of the specific motifs within the *ACVRL1 *promoter region. It can be speculated that the Sp1 binding to the starting transcriptional machinery complex is needed to ensure a transcription driven by Sp1 using different TSSs.

Because the closest region to the + 1 TSS is framed by the construction -284/+59 pALK1 and its transcriptional levels are similar to those of the -1,035/+210 pALK1 vector, probably the functional involvement of Sp1 for *ACVRL1 *transcription initiation is critical in this region. When searching the Sp1 consensus sites within this region, an interesting double site in the -89/-56 region was found. This Sp1 rich fragment contains two putative Sp1 consensus sites (-84/-78 and -67/-62) and, as shown in EMSA experiments, both sites are functional in binding to Sp1.

One key factor that modulates the Sp1 transcription dependency is the degree of methylation of the CpG islands contained within the Sp1 consensus elements. This is consistent with the observation that DNA methylation may interfere with the binding of Sp1 to DNA [41]. In fact, we found that methylation of the -89/-56 Sp1 probe of *ACVRL1 *rendered this consensus motif inactive for Sp1 binding. It is well established that DNA methylation of CpG islands is an important mechanism for transcriptional regulation of multiple genes in mammals [42]. However, most of these findings are related to proto-oncogenes, meaning that the hypermethylation is a protective mechanism for controlling the transcriptional switch to oncogenes. On the other hand, hypomethylation of tumor suppressor genes is a control mechanism for assuring its transcriptional rate. However, there are not many reports of genes non-related to cancer that could be controlled by their methylation state. For example, demethylation of certain promoters is involved in the return of somatic cells to previous undifferentiated stages of their cell lineage, and in the reprogramming to a new differentiating pathway in response to certain stimuli [43]. In some genes, cytosine methylation of Sp1 sites has been reported as a marker of organ-specific expression and as a specific regulator of the expression [44]. That could be the case of *ACVRL1*, in which demethylation of DNA in endothelial cells leads to a marked increase of *ACVRL1 *transcription, potentially because demethylation of CpG islands within the Sp1 sites is involved in *ACVRL1 *basal transcription.

ALK1 expression has been reported in several cell types, but its major roles are related to its predominant expression in endothelium. The ALK1 specific presence at the endothelial cell surface is tightly involved in the regulation of the TGF-β signalling pathway in balance with other type I TGF-β receptors [35]. The endothelial specific expression may be explained by the presence within the proximal promoter region of *ACVRL1 *of consensus motifs for transcription factors (Ets, NFκB, Sp1 and KLFs) also shared by other endothelial specific genes such as *ENG*, *PECAM1 (*Platelet Endothelial Cell Adhesion Molecule 1), *VEGFR2 (*Vascular Endothelial Growth Factor Receptor *2)*, *CDH5 *(Cadherin 5), *eNOS/NOS-3*, or *TIE2 *(Tyrosine kinase with Immunoglobulin-like and EGF-like domains 2) [30,31,45-49]. In addition, the involvement of distal regulatory regions in the human *ACVRL1*, such as the one described in mouse that confers arterial endothelium-specificity, can not be excluded [50]. Whether the degree of *ACVRL1 *methylation correlates with the endothelial specific expression of ALK1, remains to be explored.

## Conclusions

Novel *ACVRL1 *transcripts have been identified, the *ACVRL1 *promoter has been characterized and its regulation by Sp1 has been demonstrated. Furthermore, a close dependence between *ACVRL1 *expression and the CG methylation degree was found. Future experiments to identify other trans-acting or trans-repressing factors in *ACVRL1 *regulation remain to be addressed.

## Methods

### 5'Rapid Amplification of cDNA Ends (5'RACE)

RNA from human umbilical vein endothelial cells (HUVEC) was extracted and purified with RNeasy (Qiagen, Hilden, Germany) following the manufacturer's protocol. 5' RNA-Ligase-Mediated rapid amplification of cDNA ends (RLM-RACE) was carried out according to the manufacturer's instructions (Ambion Inc., Austin, TX, USA). Prior to the reverse transcription-polymerase chain reaction (RT-PCR), total RNA was treated with calf intestinal phosphatase (CIP) to remove the 5'phosphate from all RNA species except intact mRNA bearing the the 5' CAP structure. Then, tobacco acid pyrophosphatase (TAP) was used to remove the cap structure from mRNA leaving a 5'-phosphate exposed and available for the subsequent ligation. The use of a CIP/TAP-pre-treatment increases 5'RACE selectivity by avoiding the presence of false PCR amplification products, by selecting the complete mRNAs from the total RNA population. Next, a synthetic RNA oligonucleotide was added and ligated to the CIP/TAP treated RNA and the chimeric RNA was reverse transcribed using random primers. RT-PCR was performed with the avian-myeloblastosis virus (AMV) reverse transcriptase (RT) kit (Roche Diagnostics, Mannheim, Germany). The resulting cDNA was used as a template for two nested PCR. PCR was performed using different forward oligonucleotides complementary to the 5'RACE adapter and two reverse *ACVRL1*-specific primers, outer and inner, respectively. The sequences of the primers are indicated in Table 2. PCR was performed using an annealing temperature of 58°C, and with HotMaster Taq polymerase (Eppendorf, Westbury, NY, USA).

**Table 2:** Primers and probes used

5'RACE RNA-adapter	GCUGAUGGCGAUGAAUGAACACUGCGUUUGCUGGCUUUGAUGAAA
**5'RACE PRIMERS**	

5'RACE-adapter outer primer Fwd	5'-GCTGATGGCGATGAATGAACACTG-3'
*ACVRL1 *specific outer primer Rev	5'-GGGAGAGTCCAGTCTCATCCTGAA-3'
5'RACE-adapter inner primer Fwd	5'-CGCGGATCCGAACACTGCGTTTGCTGGCTTTGATG-3'
*ACVRL1 *specific inner primer Rev	5'-GCAGTAGTGGTTGACGAACTCGGT-3'

**pALK1 CLONING PRIMERS**	

Cloning primer (-898/-880) Fwd	5'-GAAGCCATTCTGCTTCCC-3'
Cloning primer (-587/-569) Fwd	5'-ACAAATGGGGGACGAAGG-3'
Cloning primer (-422/-404) Fwd	5'-AAGGATAGGTAGTGTCCC-3'
Cloning primer (-284/-263) Fwd	5'-ACGTTGCCTACAGTCTCG-3'
Cloning primer (+42/+59) Rev	5'-ATTCCAGCGTCTTCCTGC-3'

**PCR PRIMERS**	

hALK1 primer Fwd	5'-ATCTGAGCAGGGCGACAGC-3'
hALK1 primer Rev	5'-ACTCCCTGTGGTGCAGTCA-3'
h18S primer Fwd	5'-CTCAACACGGGAAACCTCAC-3'
h18S primer Rev	5'-CGCTCCACCAACTAAGAACG-3'
hSp1 primer Fwd	5'-TGCAGCAGAATTGAGTCACC-3'
hSp1 primer Rev	5'-TTGGTACTGCTGCCACTCTG-3'
hGAPDH primer Fwd	5'-AGCCACATCGCTCAGACAC-3'
hGAPDH primer Rev	5'-GCCCAATACGACCAAATCC-3'
hId1 primer Fwd	5'-GCTGCTCTACGACATGAACG-3'
hId1 primer Rev	5'-CTCCAACTGAAGGTCCCTGA-3'

**ChIP PRIMERS**	
pALK1 First region primer (-1032/-1012) Fwd	5'-CCAGAAGGCTAGGACTAAGA-3'
pALK1 First region primer (-844/-864) Rev	5'-CTCCCTGGAACTCTGCTGAC-3'
pALK1 Second region primer (-864/-844) Fwd	5'-GTCAGCAGAGTTCCAGGGAG-3'
pALK1 Second region primer (-662/-682) Rev	5'-TTAGCCCTGAGGATGGTTTG-3'
pALK1 Third region primer (-510/-490) Fwd	5'-AAAACGCATCTGGATTTTGC-3'
pALK1 Third region primer (-260/-280) Rev	5'-GAGCCGAGACTGTAGGCAAC-3'
pALK1 Fourth region primer (-200/-180) Fwd	5'-CCCACGGCCTGAGTCCAAGG-3'
pALK1 Fourth region primer (+20/+40) Rev	5'-GGCCCAGCTCCTCCACTCC-3'
pEPO primer Fwd (-66/-42)	5'-AGCCTCTCCCCCACCCCCAGCCCGGCG-3'
pEPO primer Rev (+31/+9)	5'-CAGCCCGCGAGTACTCACCGTG-3'

**EMSA PROBES**	
Sp1 sites WT pALK1 (-89/-56) Fwd	5'-CTGAGGGGCTGGGAGCGGCGCGGGGGTGGGTCCC-3'
Sp1 sites WT pALK1 (-89/-56) Rev	5'-GGGACCCACCCCCGCGCCGCTCCCAGCCCCTCAG-3'
Sp1 site MutA -84/-78 pALK1 Fwd	5'-CTGAG**TTTT**T**T**GGAGCGGCGCGGGGGTGGGTCCC-3'
Sp1 site MutA -84/-78 pALK1 Rev	5'-GGGACCCACCCCCGCGCCGCTCC**A**A**AAAA**CTCAG-3'
Sp1 site MutB -67/-62 pALK1 Fwd	5'-CTGAGGGGCTGGGAGCGGCGCG**TTTTTTT**GTCCC-3'
Sp1 site MutB -67/-62 pALK1 Rev	5'-GGGAC**AAAAAAA**CGCGCCGCTCCCAGCCCCTCAG-3'
pENG (-50 and -24 sites) Fwd	5'-GCAGGCGGCCTGGGCCCAGCCCCTTCTC-3'
pENG (-50 and -24 sites) Rev	5'-GAGAAGGGGCTGGGCCCAGGCCGCCTGC-3'
pALK1 (-823/-795) Fwd	5'-TAAAGGTCCATTTGCTGGGGTGGGGGCCC-3'
pALK1 (-823/-795) Rev	5'-GGGCCCCCACCCCAGCAAATGGACCTTTA-3'
AATT probe Fwd	5'-AAAAAAAAAAAAAAAAAAAAAAAAAAAAAAAAAA-3'
AATT probe Rev	5'-TTTTTTTTTTTTTTTTTTTTTTTTTTTTTTTTTT-3'

Note: Characters in bold/underlined in EMSA probes indicate mutated nucleotides.

### Real time PCR

For quantitative analysis, total RNA was isolated from cells using the RNeasy kit (Qiagen) and was reverse-transcribed using AMV reverse transcriptase (Roche Diagnostics). The resultant cDNA was used as a template for real time PCR performed with the primers shown in Table 2 using the iQ SyBR-Green Supermix (BioRad, Hercules, CA, USA). Amplicons were detected using an iQ5 real time detection system (BioRad). Transcript levels were normalized to 18S levels. Triplicates of each experiment were performed.

### Cloning of the *ACVRL1 *promoter fragment

A genomic *ACVRL1 *fragment corresponding to the 5'-proximal region upstream the (+ 1) TSS was cloned into *Sac*I/*Xho*I sites of the reporter vector pGL2-*luc *containing the promoterless firefly luciferase gene (Promega, Madison, WI, USA). This construct comprises 1,244 bp and extends from the position 50,586,434 to 50,587,679 of the contig (GenBank: NC_000012.10. Reference Assembly). The construct was checked by sequencing with pGL primers 1 (Forward) and 2 (Reverse) (Promega) and the resulting sequence was identical to that of the GenBank.

### Alignment among different species and *in silico *analysis of the *ACVRL1 *promoter

The human *ACVRL1 *promoter sequence was compared with the orthologous promoters in a set of animal species. The sequences to be compared were chosen by identifying the theoretical + 1 TSS and then aligning the human -1,035/+210 bp region. The accession numbers of the different contigs and the sequences used are: *Mus musculus *[GenBank:NT_039621] 62,243,937 - 62,245,182, plus strand; *Rattus norvegicus *[GenBank:NW_047784] 6,594,766 - 6,596,011, plus strand; *Bos taurus *[GenBank:NW_001495018] 123,378 - 122,133, minus strand; *Canis familiaris *[GenBank:NW_76284] 3,087,613-3,086,368, minus strand; *Pan troglodytes *[GenBank:NC_006479] 2,296,228 - 2,294,983, minus strand; *Pongo pygmaeus *[EMBL:413.52] 51,613,841 - 51,615,086, plus strand; and *Macaca mulatta *[GenBank:NW_001096621] 1,104,215 - 1,105,460, plus strand. For the *in silico *analysis of the putative response elements, we used the Genomatix MatInspector software tool: http://www.genomatix.de/products/MatInspector. The multiple sequence alignment was performed with the ClustalW2 software http://www.ebi.ac.uk/Tools/clustalw2, which generates a similarity score (values from 1 to 100) and provides a consensus proposal. Weblogo tool http://weblogo.berkeley.edu/logo.cgi was very useful to make the graphic representation of these consensus regions.

### Generation of 5'deleted fragments of the human *ACVRL1 *promoter cloned into pGL2

Four different constructs with serial deletions of the *ACVRL1 *promoter were generated by PCR amplification, using primers designed to generate fragments with differences of ~150 bp between each other. All the sequences of the cloning primers are given in Table 2. Forward primers were at positions: -898/-880, -587/-569, -422/-404 and -281/-263. In all cases, the reverse primer was at positions: + 42/+59. The amplified fragments were: pALK1 -898/+59 (957 bp); pALK1 -587/+59 (646 bp); pALK1 -422/+59 (481 bp) and pALK1 -284/+59 (343 bp). The resulting products were purified and cloned into pCR2.1-TOPO-TA vector (Invitrogen, Carlsbad, CA, USA). Then, they were digested using *Sac*I and *Xho*I flanking restriction sites, inserted in pGL2-*luc *and checked by sequencing with commercial pGL primers 1 and 2 as described above.

### Cell culture

*Drosophila *Schneider S2 cells were grown in *Drosophila*-enriched Schneider's (DES) insect medium (Sigma Aldrich, St Louis, MO, USA) supplemented with 10% fetal bovine serum (FBS), 0.1 μg/μl gentamicin and 2 mM L-glutamine. The human microvasculature endothelial cell line HMEC-1 was grown on 0.2% gelatin (Sigma Aldrich) pre-coated plates in MCDB-131 medium (Gibco, Paisley, UK) supplemented with 10% FBS, 2 mM L-glutamine, 1 ng/mL Epidermal Growth Factor (EGF) and 1 μg/ml hydrocortisone. Primary HUVEC were grown in EBM2 medium supplemented with EGM2 (Lonza, Walkersville, MD, USA) and containing 10% FBS. Human epithelial embryonic kidney HEK293T cells were cultured in Dulbecco's modified Eagle's medium (DMEM) with 10% FBS. All the media were supplemented with both 100 U/ml penicillin-streptomycin.

### DNA transfections and luciferase assays

Transfections of HEK293T, HMEC-1 and Schneider S2 cells were performed using the Superfect Reagent (Qiagen) following the commercial instructions. When required, transfected cells were treated for 3 hours with 1 ng/ml of TGF-β1 (R&D Systems, Minneapolis, MN, USA) or 16 hours with 0.5 ng/ml of BMP9 (R&D Systems) in the presence of 2% FBS. The expression vector pCIneo-Sp1 was used to transfect mammalian cells, whereas pPac-Sp1 was used to transfect *Drosophila *Schneider S2. The corresponding empty vectors were used as controls. Forty eight hours after transfection with the pGL2 reporter vectors, cells were harvested and the luciferase activities were determined in a TD-20/20 luminometer (Promega). In all cases, the pCMV-βGal vector was included in the transfections and its transcriptional activity was measured as an internal control. After normalization, the activity of the reporter constructs was referred to the basal activity as fold induction or as percentages with respect to controls.

### Sp1 knock down

The human Sp1 small interfering ribonucleic acid (siRNA) was obtained from Santa Cruz Biotechnology (sc-29487, Santa Cruz, CA, USA). HEK293T cells were transfected with 5 pmoles of siRNA hSp1 or scrambled siRNA, using lipofectamine 2000 (Invitrogen, Carlsbad, CA, USA). Twenty four hours later, the pALK1 reporter construct and the Renilla normalization plasmid were transfected, using the Superfect Reagent (Qiagen). Luciferase activity was measured 24 hours after DNA transfection with the Dual Luciferase Assay System (Promega). Values were normalized to Renilla activity and values were referenced to the basal activity (100%) in each case. Lysates from these cells at 48 hours post-transfection were analyzed by semiquantitative RT-PCR using the primers indicated in Table 2 and by western blot using the rabbit polyclonal antibody anti-Sp1 (PEP2, SC-59, Santa Cruz Biotechnology) and monoclonal mouse antibody anti-β-actin antibody AC15 (A1978, Sigma Aldrich).

### Chromatin immunoprecipitation (ChIP)

ChIP was performed with ChIP-IT Express kit (Active motif, Rixensart, Belgium), following the manufacturer's instructions. Briefly, HUVEC were grown to confluence and subsequently fixed with 1% formaldehyde. Cells were scraped in the presence of PMSF (phenylmethylsulphonyl fluoride) and lysed. Nuclei were separated using a dounce homogenizer and digested with enzymatic shearing cocktail for 15 min. One aliquot of this sheared chromatin was used as "input chromatin" and the rest was incubated with protein G magnetic beads and rabbit polyclonal antibody against human Sp1 PEP2 (SC-59, Santa Cruz Biotechnology) on a rolling shaker for 4 hours at 4°C. The positive control was incubated with rabbit polyclonal anti-human Histone 3 (ab8580, Abcam, Cambridge, MA, USA) and the negative control with serum anti-human IgG. Protein G magnetic beads bound to the immune complexes were pelleted, washed and bound proteins were eluted with the elution buffer provided with the kit. Then, the crosslinking was reversed and samples were incubated with proteinase K during 1 h at 37°C. Primers used for PCR were selected by mapping the whole promoter sequence, separated into four regions. The first region encompasses from -1,032 to -844 (188 bp); the second region from -864 to -662 (202 bp); the third region from -510 to -260 (250 bp) and the fourth region from -200 to + 40 (240 bp). Sequences of the four couples of primers are indicated in Table 2. For negative and positive control PCR, primers from ChIP-IT control kit human (Cat # 53010, Active motif) were used (data not shown). Measurement of the Sp1 binding was carried out using the following ratio of band intensities: (Sp1-IgG)/PInput.

### Electrophoretic mobility shift assay (EMSA)

For the radiolabelled probe, the oligonucleotides were designed in the region -89/-56 of the *ACVRL1 *promoter, which includes two Sp1 consensus sites flanking a Krüppel-like factor-6 (KLF6) site. Competition experiments were carried out with five cold probes: i) -89/-56 wild type (WT); ii) -89/-56 with the site -84/-78 mutated; iii) -89/-56 with the site -67/-62 mutated; iv) -823/-795, containing consensus binding sites for other transcription factors, none of them homologous to Sp1 motifs; and v) AATT, an artificial competitor, double stranded poly dA-poly dT sequence, unrelated to the *ACVRL1 *promoter. The corresponding sequences are shown in Table 2. Probes were prepared by annealing complementary synthetic oligonucleotides followed by end labelling with [γ^32^P]dATP and T4 polynucleotide kinase. Nuclear extracts from HeLa cells were obtained from Promega (Cat # E3521). Approximately 5 ng (100,000 cpm) of the respective probe was incubated with 10 μg of nuclear extract and 2 μg/reaction of poly (dI-dC) for 30 min on ice. For competition experiments, a 100-fold excess of unlabeled double-stranded oligonucleotide was added. For supershift assays, protein extract and 1 μg of commercial antibody were preincubated for 60 min on ice prior to the addition of the remaining components of the binding reaction. Rabbit polyclonal antibodies anti-Sp1 (PEP2, SC-59), anti-Sp3 (SC-644) and anti-NFκB (Nuclear Factor kappa-light-chain-enhancer of activated B cells) (H-119, SC-7178) were purchased from Santa Cruz Biotechnology.

Positive control binding reactions were performed with a probe designed for the *ENG *promoter (-50/-24 Sp1 sites), which strongly binds Sp1 [31]. Binding reactions were separated by nondenaturing 6% polyacrylamide gel electrophoresis in Tris-Borate-EDTA buffer at 4°C. Gels were dried, and visualized by autoradiography. EMSAs were repeated at least three times with similar results.

### Treatment of cells with the demethylating agent 5'-aza-2'-deoxycytidine

CpG islands were detected using the software tool CpGplot http://www.ebi.ac.uk/Tools/emboss/cpgplot/index.html. HUVEC, HMEC-1, and HEK293T cells were treated with 5'-aza-2'-deoxycytidine (5-aza-dC; Sigma Aldrich) at a final concentrations of 1 μM or 5 μM for 7 days and the medium was changed every second day, according to Butta et al., [51]. Finally, cells were lysed, total RNA was extracted with RNeasy kit (Qiagen) and the ALK1/Id1 ratio was measured by real time PCR. Primers used are shown in Table 2.

For functional experiments, after a one week of treatment with 5 μM 5-aza-dC, HMEC-1 cells were transfected with the specific reporter of the ALK1 pathway p(BRE)_2_-*luc *(BMP-responsive firefly luciferase reporter), which contains a small sequence with two copies of the regions (-1052/-1032)/(-1105/-1080) of the Id1 promoter [52].

### *In vitro *methylation of promoters

Constructs -1,035/+210, -898/+59, -587/+59, -422/+59 and -284/+59 of *ACVRL1 *promoter and the reporter plasmids of Id1 promoter (pGL2-pId1) and the rat minimal prolactin promoter region -36 to + 37 surrounding a TATA box, (pXP2-TATAbox) [53] were methylated *in vitro *by the CpG methylase M.*Sss*I (New England Biolabs, Ipswich, MA, USA) in the absence or presence of the substrate S-adenosylmethionine. The methylation status was checked by digestion with the restriction enzyme *Hpa*II, which digests the target sequence CCGG but only when it is unmethylated (data not shown). Cells were transfected with 100 ng of the indicated mock-methylated or methylated *ACVRL1 *promoter reporter, 100 ng of pCIneo-Sp1 and with Renilla reporter vector. Luciferase activity was measured after 48 hours and normalized to Renilla activity. For electrophoretic mobility shift assays, the cold probe (-89/-56) of *ACVRL1 *promoter was subjected to *in vitro *methylation with the CpG methylase M.*Sss*I prior to competition with the radiolabelled probe.

### Statistics

Data were subjected to statistical analysis and results are shown as mean ±SD. Differences in mean values were analysed using Student's t-test. In the figures, the statistically significant values are marked with asterisks (*p < 0.05; **p < 0.01; ***p < 0.005; ns = not significant).

## Abbreviations

ACVRL1: activin-A receptor type II-like kinase 1; ALK1: Activin receptor-Like Kinase 1; 5-aza-dC: 5'-aza-2'-deoxycytidine; BMP9: Bone Morphogenetic Protein-9; EMSA: Electrophoretic mobility shift assay; ENG: Endoglin; eNOS/NOS-3: Endothelial Nitric Oxide Synthase; Ets: E26-Transformation-specific Transcription Factor; FBS: Fetal Bovine Serum; HHT: Hereditary Hemorrhagic Telangiectasia; HIF: Hypoxia Inducible Factor; KLF6: Krüppel-Like Factor 6; NFκB: Nuclear Factor of kappa light polypeptide gene enhancer in B-cells; 5'RACE: 5'Rapid Amplification of cDNA ends; RXR: Retinoid X Receptor; SBE: Smad Binding Element; Sp1: Specificity Protein 1; TGF-β: Transforming Growth Factor-β; TSS: Transcriptional Start Site.

## Authors' contributions

EMG-M, FJB, CB and LMB conceived, designed and coordinated the study and wrote the manuscript. All authors participated in analysis of the results. All authors have read and approved the final manuscript.
